# Membrane Proteins and Proteomics of Cronobacter sakazakii Cells: Reliable Method for Identification and Subcellular Localization

**DOI:** 10.1128/aem.02508-21

**Published:** 2022-04-18

**Authors:** Jiří Novotný, Barbora Svobodová, Jiří Šantrůček, Ladislav Fukal, Ludmila Karamonová

**Affiliations:** a Department of Biochemistry and Microbiology, University of Chemistry and Technology, Prague, Czech Republic; University of Helsinki

**Keywords:** *Cronobacter*, food pathogen, membrane proteomics, cell subfractionation

## Abstract

Members of the genus *Cronobacter* are responsible for severe infections in infants and immunosuppressed individuals. Although several virulence factors have been described, many proteins involved in the pathogenesis of such infections have not yet been mapped. This study is the first to fractionate Cronobacter sakazakii cells into outer membrane, inner membrane, periplasmic, and cytosolic fractions as the basis for improved proteome mapping. A novel method was designed to prepare the fractionated samples for protein identification. The identification was performed via one-dimensional electrophoresis-liquid chromatography electrospray ionization tandem mass spectrometry. To determine the subcellular localization of the identified proteins, we developed a novel Python-based script (Subcelloc) that combines three web-based tools, PSORTb 3.0.2, CELLO 2.5, and UniProtKB. Applying this approach enabled us to identify 1,243 C. sakazakii proteins, which constitutes 28% of all predicted proteins and 49% of all theoretically expressed outer membrane proteins. These results represent a significant improvement on previous attempts to map the C. sakazakii proteome and could provide a major step forward in the identification of *Cronobacter* virulence factors.

**IMPORTANCE** *Cronobacter* spp. are opportunistic pathogens that can cause rare and, in many cases, life-threatening infections, such as meningitis, necrotizing enterocolitis, and sepsis. Such infections are mainly linked to the consumption of contaminated powdered infant formula, with Cronobacter sakazakii clonal complex 4 considered the most frequent agent of serious neonatal infection. However, the pathogenesis of diseases caused by these bacteria remains unclear; in particular, the proteins involved throughout the process have not yet been mapped. To help address this, we present an improved method for proteome mapping that emphasizes the isolation and identification of membrane proteins. Specific focus was placed on the identification of the outer membrane proteins, which, being exposed to the surface of the bacterium, directly participate in host-pathogen interaction.

## INTRODUCTION

*Cronobacter* spp. (formerly Enterobacter sakazakii) are Gram-negative facultative anaerobic bacteria belonging to the *Enterobacteriaceae* family. The genus *Cronobacter* was established after extensive taxonomic changes in 2008 and currently includes seven species (1, 2). According to FAO/WHO, all *Cronobacter* species are potentially pathogenic (3). In particular, they are opportunistic foodborne pathogens especially associated with the contamination of powdered infant formula. The consumption of contaminated formula by neonates, particularly premature neonates, can result in life-threatening necrotizing enterocolitis and meningitis (4). Based on clinical prevalence supported by multilocus sequence typing (MLST), Cronobacter sakazakii is the most frequently found species in clinical samples, with strains of clonal complex 4 involved in the most severe meningitis contracted by neonates. Despite the increasing number of studies using whole-genome sequencing to propose various C. sakazakii virulence factors, none of these factors has been uniquely linked to *Cronobacter* pathovars (5).

Although comparative genomics can recognize strain variability, proteomics provides essential information on how the genotype manifests in the phenotype of organisms. Proteins are powerful agents that execute the expression of genetic information and display bacterial adaptation to the host environment (6). In particular, membrane proteins are the most common bacterial virulence factors because they represent the key point at which bacteria interact with the environment, host cells, and immune system. Furthermore, because they enable bacteria to enter and proliferate in host cells, they contribute to disease pathogenesis (7).

The identification of membrane proteins from pathogenic bacteria remains a challenging task in proteomic studies due to their heterogeneity, hydrophobicity, and relatively low abundance (8). In fact, membrane proteins represent about 20 to 30% of all encoded bacterial proteins, yet they are still underinvestigated in proteome research (9). This is in large part due to their poor solubility in aqueous systems, which makes it necessary to use detergents to mimic the natural environment of the lipid bilayer. The problem with this is that detergents can negatively influence further identification by mass spectrometry. Furthermore, the hydrophobic regions of membrane proteins resist enzymatic cleavage by specific proteases. Once cleaved, the peptides from the membrane regions are usually rich in hydrophobic amino acids and, thus, become less suitable for ionization and fragmentation by mass spectrometry (10).

Few proteomic studies have targeted the *Cronobacter* membrane and whole-cell (WHC) proteins, and those that have have adopted different approaches. In 2007, Riedel et al. fractionated C. sakazakii cells into WHC and surface-associated (SF) proteins to monitor those expressed under osmotic stress conditions (11). To yield WHC proteins, the authors used cell lysis by sonication, but while this procedure isolates WHC proteins, it has a preference for cytosolic (C) proteins rather than for hydrophobic membrane proteins. To yield SF proteins, they used acid-base extraction, but the acid-base extraction of membrane proteins is typically heavily contaminated with C and inner membrane (IM) proteins and, thus, not satisfactory for the isolation of outer membrane (OM) proteins. Subsequently, the WHC and SF proteins were separated by two-dimensional electrophoresis (2DE) and identified by matrix-assisted laser desorption ionization–time of flight mass spectrometry (MALDI-TOF MS) (12). Generally, the former is not recommended for membrane proteins because these highly hydrophobic proteins often precipitate at their isoelectric points during isoelectric focusing, while the latter is less sensitive to low-abundance membrane proteins (13, 14). Carranza et al. used a very similar approach to identify the proteome of *C. turicensis* z3032 but with minor improvements based on the use of Triton X-100 and 3-[(3-cholamidopropyl)-dimethylammonio]-1-propanesulfonate (CHAPS) detergents to extract WHC and SF proteins, respectively (15). In addition, the procedure was extended to include the isolation of extracellular (EC) proteins. To map the EC and the SF proteins, the more suitable 1DE analysis was used; the SF proteins were identified by highly sensitive liquid chromatography electrospray ionization tandem MS (LC-ESI-MS/MS) and WHC and EC proteins by 2D-LC-MALDI-TOF/TOF. However, only 19% of all theoretically expressed proteins were identified, indicating that there is considerable scope for improvement in this protein preparation technique.

More recently, several other studies have investigated C. sakazakii strains (in all cases, one virulent and one attenuated) by focusing solely on WHC or whole-membrane proteins. The WHC proteins were obtained from a commercial kit or by cell lysis using liquid nitrogen (16, 17), while the membrane proteins were extracted by phase separation using Triton X-114 (18). Although the researchers successfully identified several upregulated proteins in more virulent strains, such as EnvZ, LptE, MdtD, and OsmYb, all studies used the less sensitive combination of 2DE and MALDI-TOF/TOF for protein identification.

Two studies have focused on *Cronobacter* OM proteins obtained by treatment with *N*-lauroylsarcosine (sarcosyl) (19, 20). In 2011, Jaradat et al. used this approach to analyze *C. muytjensii* OM proteins and their antigenic properties. Using 2DE-MALDI-TOF/TOF, they discovered that OM proteins are much conserved in the *Enterobacteriaceae* family and promote antigenic cross-reactivity between genera (19). More recently, Aldubyan et al. studied the occurrence of OM proteins in highly invasive and low-invasiveness strains of *C. malonaticus* (20). Their method combined sarcosyl treatment and ultracentrifugation for the isolation of OM proteins and 1DE-LC-ESI-MS/MS as well as 2DE-MALDI-TOF/TOF for identification.

They showed that, unlike the low-invasiveness strains, the highly invasive strains possessed several flagellar proteins, but they were only able to identify a few OM proteins (20). Thus, despite the fact that several proteomic studies have attempted to elucidate *Cronobacter* proteins, there remains a deficit of knowledge regarding the proteins associated with the periplasm (PP), IM, and OM itself.

## RESULTS

### *In silico* prediction of subcellular localization.

To determine the subcellular localization of proteins as accurately as possible, a Subcelloc script that utilizes the PSORTb 3.0.2 and CELLO 2.5 web servers, together with the UniProtKB database, was developed. Applying this script, the final protein localization (EC, OM, PP, IM, and C) is determined only if at least two of the three localization results match. Proteins that are only localized by one of the three localizations (the other two marked the proteins as unknown) are listed as unknown (N). If the final localization cannot be determined but the protein is not categorized as N, the script allocates this protein to the more attention needed (MAN) or membrane (MEMBR) category. Allocation to the MAN category means that the script cannot decide on the exact protein localization (prediction software shows different results); allocation to the MEMBR category means that most likely it is not a cytosolic protein, because the prediction software results do not match but are associated with membranes. Furthermore, the script provides information about the presence of a signal peptide (SP) using the SignalP 5.0 web server and of transmembrane helices (TMHs) using the TMHMM 2.0 web server. C. sakazakii BAA-894 from UniProt was selected for the *in silico* subcellular localization evaluation based on its predicted protein localization, as shown in Table 1.

**TABLE 1:** *In silico* prediction of subcellular localization of C. sakazakii strain BAA-894^*a*^

Protein	Result by:			
	PSORTb	CELLO	UniProtKB	Subcelloc
EC	54	118	5	24
FLAG			39	39
OM	85	150	82	79
PP	150	568	43	136
IM	1,032	798	344	807
MEMBR			645	219
C	1,797	2,787	468	1,748
MAN				240
MULTI	98			
N	1,205		2,795	1,129
Total	4,421	4,421	4,421	4,421

a EC, extracellular proteins; FLAG, flagellar proteins; OM, outer membrane proteins, PP, periplasmic proteins; IM, inner membrane proteins; MEMBR, membrane proteins; C, cytosolic proteins; MAN, more attention needed proteins; MULTI, proteins with multiple possible localization; N, proteins with unknown localization. Subcellular protein localization was predicted by web-based tools PSORTb 3.0.2, CELLO 2.5, UniProtKB, and Subcelloc script.

A total of 4,421 proteins from the proteome of BAA-894 were analyzed *in silico*. The results showed that PSORTb could determine subcellular localization for 70% (3,118) of the proteins, with the MULTI and N categories accounting for the other 1,303. Because CELLO can only select an existing localization, prediction using this tool means identifying the localization of 100% of the proteins. UniprotKB could determine the localization of only 37% (1,626) of proteins, most of which (645) were in the ambiguous MEMBR category. Although PSORTb has the best determination capability of all three prediction tools, there is always the possibility that it can incorrectly predict subcellular localization. Thus, combining these tools in a Subcelloc script proved more effective, with our script dividing 74% (3,292) of the proteins into the created categories and characterizing just 1,129 proteins as N. Figure S1 in the supplemental material shows a Venn diagram representing the subcellular localization predicted using PSORTb, CELLO, and UniProtKB. The ambiguously localized proteins could be further analyzed using the time-consuming Basic Local Alignment Search Tool (BLAST) search to compare sequences of desired proteins with proteins from related species. However, as our study focuses on membrane proteins, BLAST was only used for proteins in the MAN and MEMBR categories as well as for N proteins that contain a β-barrel domain, a TMH, or an SP. This means that BLAST analyzed a total of 757 proteins, determining subcellular localization for 25% of those (Table 2). BLAST was only able to determine a small minority of proteins (1 of 19) in N with the BOMP category and 18 of 215 in the N with SP category. On the other hand, the specification of those in the N with TMH category was more successful (11 of 64). Thus, BLAST can be highly recommended for proteins not only in MAN and MEMBR but also in N with TMH.

**TABLE 2:** Ambiguous localization of Cronobacter sakazakii strain BAA-894 proteins by category as accurately determined by BLAST search^*a*^

Ambiguous category	No. of proteins	BLAST^*b*^
MAN	240	84
MEMBR	219	76
N with BOMP	19	1
N with SP	215	18
N with TMH	64	11
Total	757	190

a MAN, more attention needed proteins; MEMBR, membrane proteins; N with BOMP, unknown proteins with β-barrel domain; N with SP, unknown proteins with signal peptide; N with TMH, unknown proteins with transmembrane helices.

b Number of proteins for which subcellular localizations were determined using BLAST search.

The Subcelloc script with an additional BLAST search was applied to two other C. sakazakii proteomes (strains 696 and MOD1_GK958). Although all three tested proteomes possess different amounts of proteins, the number of proteins that were localized was similar, the main differences being in the N category (Table 3). Across the specified categories, there was an even distribution of proteins in the individual cell compartments of strains of the same species, although qualitatively they may be different proteins.

**TABLE 3:** *In silico* analyses of subcellular localization of proteins in three C. sakazakii strains BAA-894, 696, and MOD1_GK958^*a*^

Protein	No. of proteins in C. sakazakii strain:		
	BAA-894	696	MOD1_GK958
EC	25	27	27
FLAG	39	40	42
OM	92	84	87
PP	148	145	149
IM	900	857	904
MEMBR	146	174	128
C	1,798	1,779	1,795
N	1,273	1,532	1,049
Total	4,421	4,638	4,181

a EC, extracellular proteins; FLAG, flagellar proteins; OM, outer membrane proteins, PP, periplasmic proteins; IM, inner membrane proteins; MEMBR, membrane proteins; C, cytosolic proteins; N, proteins with unknown localization. Subcellular localization was determined using the Subcelloc script together with a BLAST search of ambiguously localized proteins.

### Subfractionation of Cronobacter sakazakii strain Cb35.

**FIG 1:**
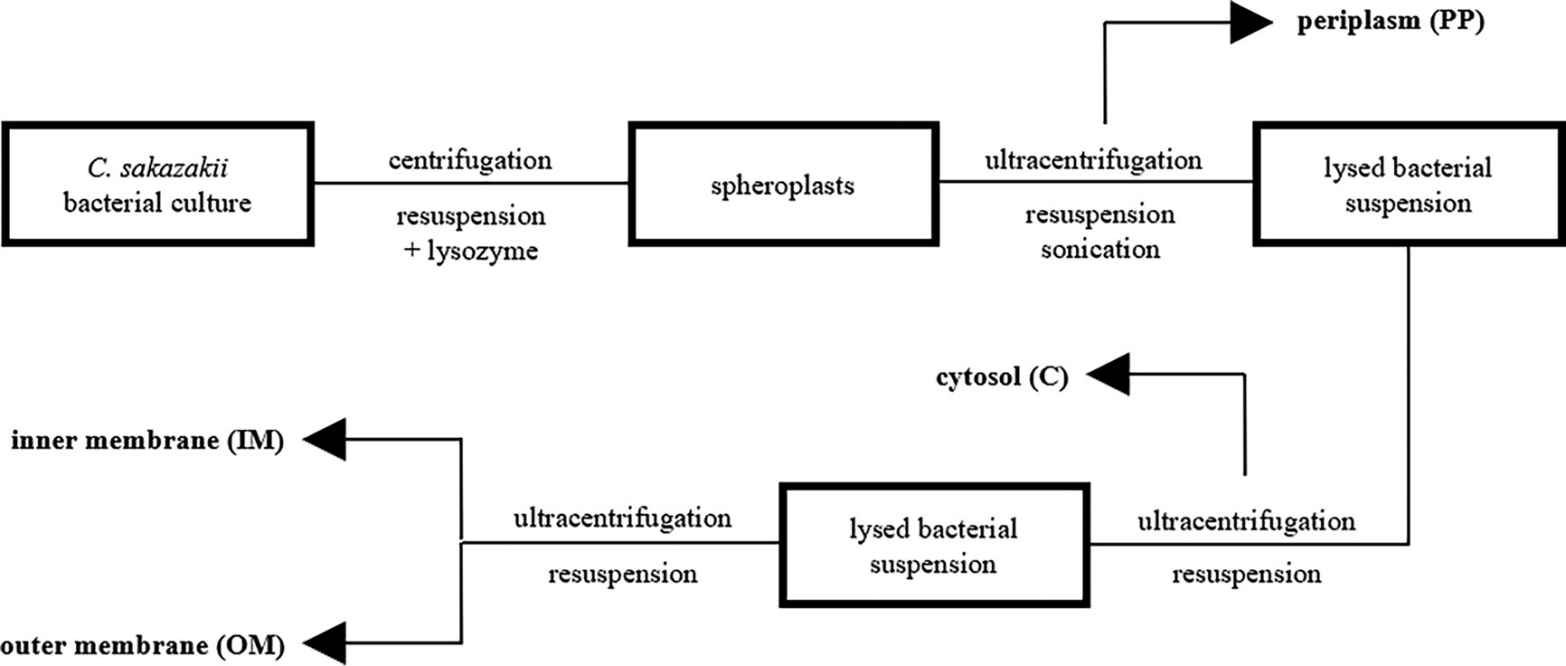
Scheme of subfractionation method (arrows refer to isolated fractions).

Proteins belonging to different cell compartments of C. sakazakii Cb35 were divided by a series of ultracentrifugation steps using different detergents (Fig. 1). The peptidoglycan of the bacterial cells was cleaved by lysozyme digestion. The PP content was released into the solution, and spheroplasts, cells encapsulated by only the inner and outer membranes, were formed. After ultracentrifugation, the PP fraction present in the supernatant was collected and stored. The pelleted spheroplasts were disrupted by ultrasound sonication. The soluble cytosol content was separated from the membranes by another ultracentrifugation. The membrane proteins remaining in the pellet were obtained step by step using Triton X-100 and ultracentrifugation.

### Preparation of isolated fractions for further MS analyses. (i) Detergent selection.

**FIG 2:**
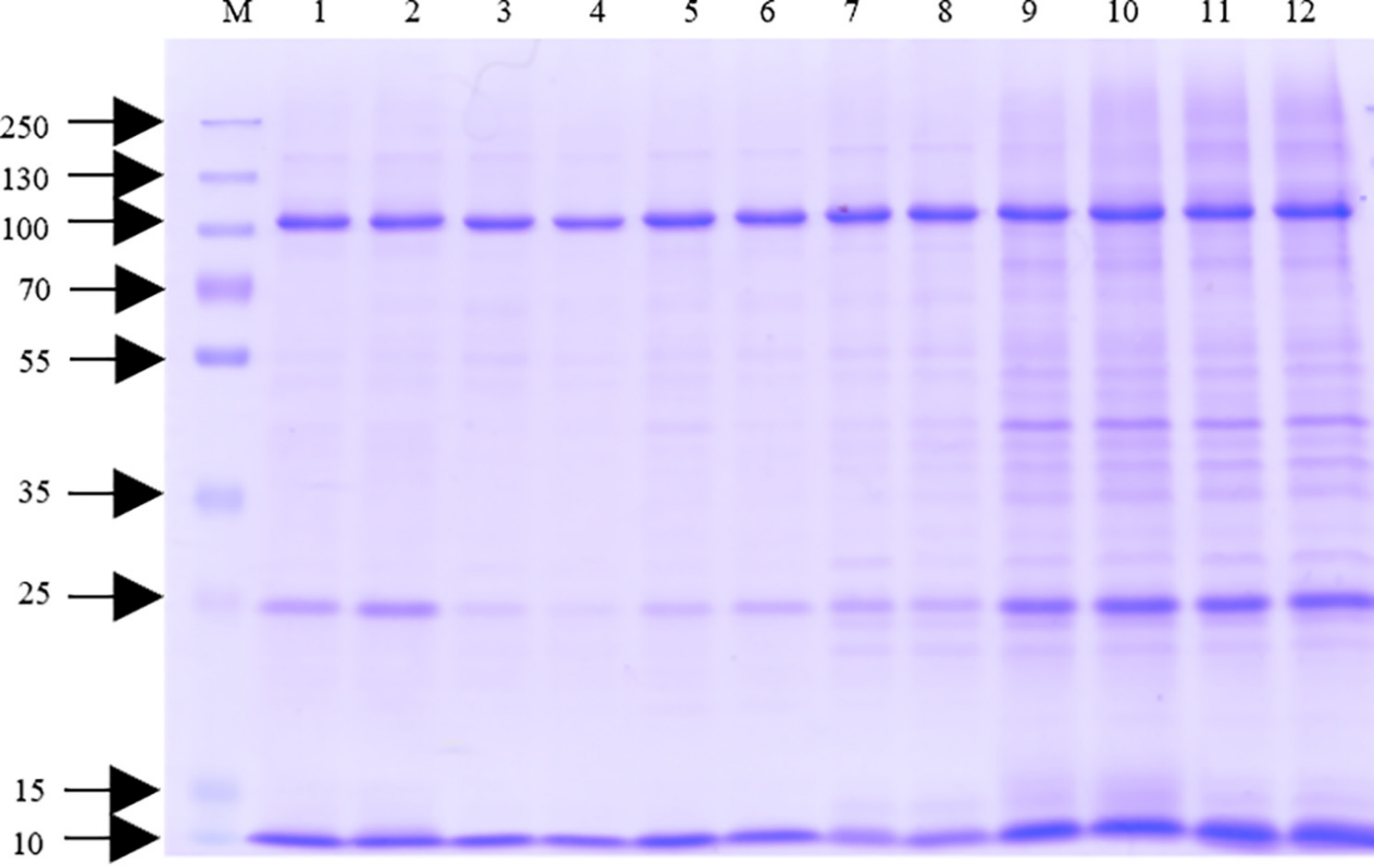
SDS-PAGE analysis of outer membrane sample of C. sakazakii strain Cb35 dissolved in various detergents. The outer membrane samples were separated by 12% SDS-PAGE. The gel was Coomassie stained. M, molecular mass standard (kDa); 1, 0.5% Triton X-100; 2, 1% Triton X-100; 3, 0.5% Tween 20; 4, 1% Tween 20; 5, 0.5% CHAPS; 6, 1% CHAPS; 7, 0.5% sodium deoxycholate; 8, 1% sodium deoxycholate; 9, 0.5% *N*-lauroylsarcosine sodium salt; 10, 1% *N*-lauroylsarcosine sodium salt; 11, 0.5% sodium dodecyl sulfate; 12, 1% sodium dodecyl sulfate.

After subfractionation, all of the isolated fractions were lyophilized and stored. The IM, PP, and C fractions were reconstituted in deionized water because all of them already contained the final subfractionation buffer. Conversely, the OM fraction, generated at the end of the subfractionation process, had only been dissolved in deionized water and, thus, needed to be dissolved in a detergent-containing buffer. Anionic (SDS, *N*-lauroylsarcosine sodium salt [sarcosyl], and sodium deoxycholate [DOC]), zwitterionic (CHAPS), and nonionic (Triton X-100 and Tween 20) detergents were tested to determine which dissolved the OM fraction most efficiently. While detergents have differing abilities to form micelles depending on their critical micelle concentration (CMC), the actual detergent concentration must not be high due to the adverse effect it would have on protein identification by mass spectrometry (21) (Table S1). The detergents were evaluated according to the following criteria: (i) the amount of proteins visualized in the samples after SDS-PAGE, (ii) the protein concentration determined by bicinchoninic acid (BCA) assay, and (iii) the amount of detergent used. The intensity of the protein zones on the Coomassie-stained SDS-PAGE gel clearly showed that the anionic detergents SDS and sarcosyl dissolved the most proteins, with no visible difference between the 0.5% and 1% solutions (Fig. 2).

The protein concentration of the OM fractions was determined using the Pierce BCA protein assay kit. Protein concentrations higher than 500 μg mL^−1^ were achieved using sarcosyl and SDS; the other detergents reached a maximum of 270 μg mL^−1^ (Table 4). In terms of CMC values (Table S1) and the subsequent MS analyses, 0.5% SDS and sarcosyl appeared to be the most suitable detergents. Because SDS is used in Laemmli buffer, sarcosyl was chosen to solubilize the OM fraction in a further preparation step.

**TABLE 4:** Protein concentration of OM fraction dissolved in various detergents determined by Pierce BCA protein assay kit

Detergent	Concn (μg mL^−1^) at:	
	0.5%	1%
Sarcosyl	529 ± 8	561 ± 4
SDS	538 ± 8	618 ± 9
DOC	164 ± 8	202 ± 6
CHAPS	268 ± 14	262 ± 8
Triton X-100	250 ± 4	269 ± 5
Tween 20	198 ± 2	165 ± 6

### (ii) Sample preparation methods.

**FIG 3:**
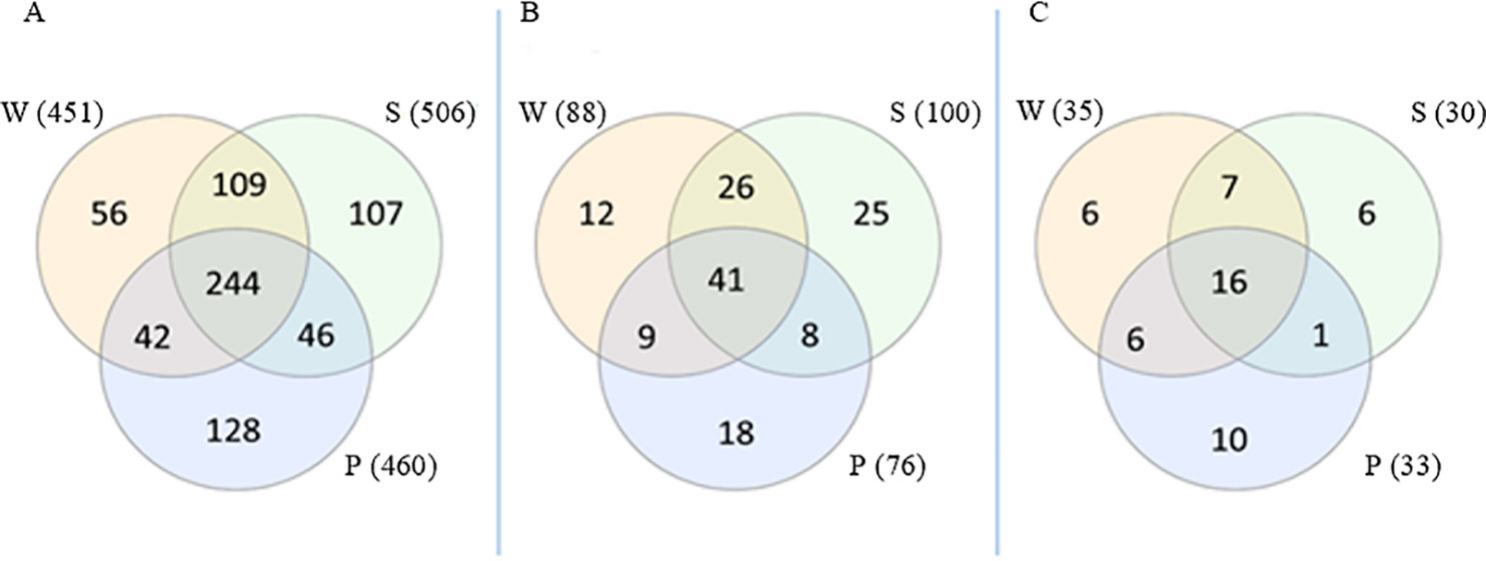
Comparison of the sample preparation procedures prior to MS analyses. Sample W was dissolved in sarcosyl, homogenized, and, without centrifugation, applied to SDS-PAGE gel. Sample S was centrifuged and only the supernatant was used. Sample P was the remaining pellet dissolved directly in Laemmli buffer. (A) Comparison of all identified proteins. (B) Comparison of proteins identified as membrane associated. (C) Comparison of proteins identified as outer membrane proteins. Subcellular localization was determined using the Subcelloc script together with a BLAST search of ambiguously localized proteins.

Three samples were prepared prior to MS analyses: sample W (whole) was dissolved in 0.5% sarcosyl, homogenized, and applied to SDS-PAGE gel without centrifugation; sample S was centrifuged and only the supernatant used; and sample P was the remaining pellet dissolved in Laemmli buffer. We wanted to know whether centrifugation of the sample affected the number of identified proteins and whether the proteins insoluble in sarcosyl remained in the pellet. When the total number of identified proteins was compared (Fig. 3A), sample S (506) had the highest overall amount of proteins while sample P (128) had the most unique proteins. Because our study is focused on membrane proteins, only proteins associated with the membrane (localized as OM, IM, PP, MEMBR, or FLAG) were used for further analyses. Of these, sample S had the highest number of both membrane (100) and unique (25) proteins (Fig. 3B). The amounts of unique OM proteins identified were comparable across all tested samples, 6 being identified in samples W and S, and 10 in sample P (Fig. 3C).

### Crucial steps affecting repeatability of the method.

**FIG 4:**
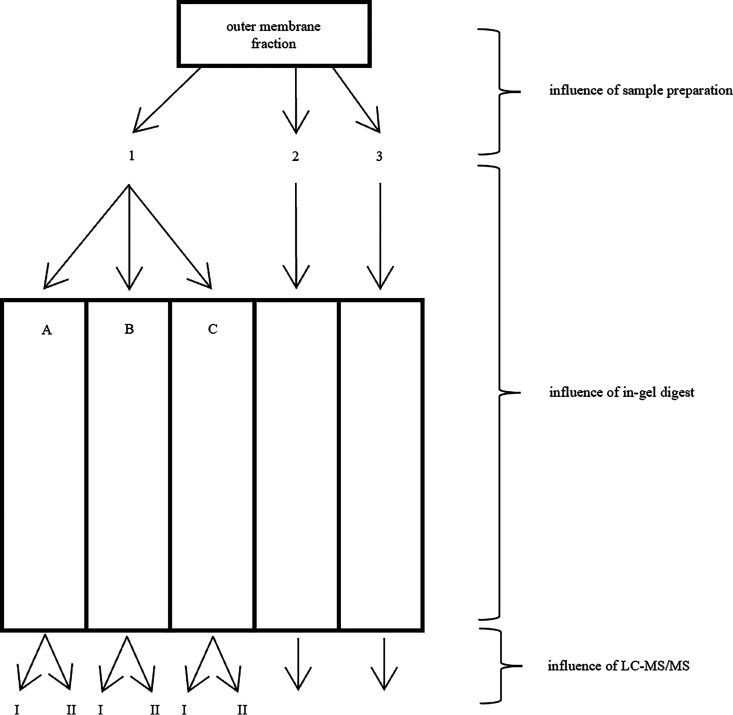
Scheme for determining the technical repeatability of the protein identification method. The outer membrane fraction was dissolved three times (marked 1, 2, and 3). Sample 1 was applied to the polyacrylamide gel three times (marked A, B, and C) and samples 2 and 3 once. Following SDS-PAGE trypsin in-gel digestion, samples A, B, and C were analyzed twice by LC-MS/MS (marked I and II).

In protein identification by mass spectrometry, qualitative differences are often associated with high sample complexity. The influence of sample preparation, in-gel digestion, and LC-MS/MS on the OM fraction of C. sakazakii Cb 35 was monitored. An OM sample was chosen because its preparation prior to MS requires additional steps, including detergent dissolution and homogenization. Figure 4 shows the three steps involved in our technical repeatability evaluation. The influence of these three crucial steps on the overall subfractionation process is summarized in Table 5. The effect of LC-MS/MS was established by comparing the proteins identified from two independent injections (marked I and II) of samples A, B, and C into the LC column. These samples (1AI and 1AII; 1BI and 1BII; and 1CI and 1CII) showed similarity ranging from 61 to 63%.

**TABLE 5:** Technical repeatability of sample preparation and protein identification^*a*^

Sample	1AI	1AII	1BI	1BII	1CI	1CII	2	3
1AI	100^*b*^							
1AII	61	100						
1BI	49	47	100					
1BII	48	51	63	100				
1CI	52	53	62	64	100			
1CII	51	52	55	65	63	100		
2	49	51	62	64	64	64	100	
3	43	44	56	57	56	56	62	100

a The OM fraction was dissolved three times (samples 1, 2, and 3); sample 1 was applied to the polyacrylamide gel three times (samples A, B, and C) and samples 2 and 3 only once. After trypsin in-gel digestion, each of samples A, B, and C was analyzed by LC-MS/MS twice (samples I and II).

b Percentage of identical proteins. Light gray-shaded numbers, influence of LC-MS/MS; dark gray-shaded numbers, influence of in-gel digestion; black numbers, influence of sample preparation. All identified proteins were compared by their UniParc protein identifiers.

Another crucial step was the SDS-PAGE in-gel digestion procedure. The OM fraction was dissolved and divided into three replicates that were subsequently loaded onto SDS-PAGE. After separation, these samples were digested by trypsin. Samples that had the same origin (sample 1) but differed in terms of their in-gel digestion and peptide purification steps were compared (samples 1A, 1B, and 1C). The similarity between these samples ranged from 47% to 65%, giving a 52% median overlap. The last crucial step was sample preparation, namely, the weighing and dissolution of the OM fraction (samples 1, 2, and 3). Here, sample repeatability resulted in a 56% median overlap. In summary, the LC-MS/MS step had the most impact on the preparation repeatability of the OM fraction, and weighing and dissolution had the least.

**FIG 5:**
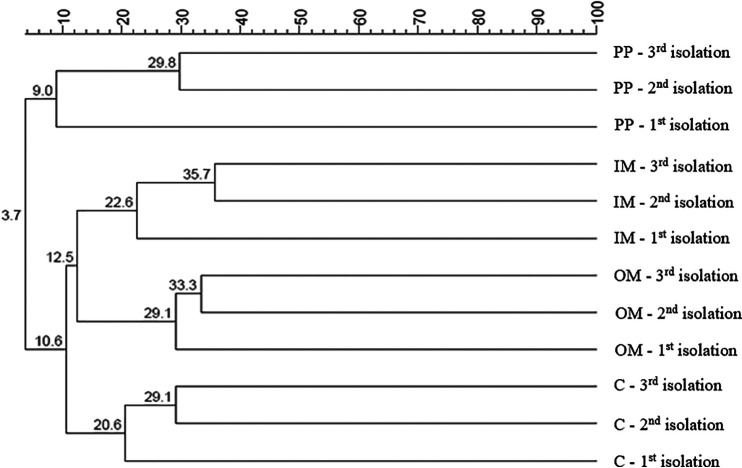
Dendrogram representing overall fraction similarity across the three independent isolations. PP, periplasmic fraction; IM, inner membrane fraction; OM, outer membrane fraction; C, cytosolic fraction. Numbers in dendrogram indicate percentage of proteins with the same sequence. Dendrogram was created with BioNumerics 7.5 software and calculated using the Jaccard correlation with a maximum limit value. The clustering method used was unweighted pair-group arithmetic mean (UPGMA) with Euclidean distance.

Due to the high variability involved when using living organisms, subfractionation was performed from three independent cultivations of C. sakazakii Cb35. The protein concentrations of the isolated fractions are shown in Table S2. UniParc protein identifiers were used to compare the similarity of the proteins in all isolated fractions (Fig. 5). The fractions were clustered according to their specific protein content; satisfactory subfractionation was indicated by different proteins predominating in different fractions. Similarity between individual subfractionation ranged from 29 to 33% for OM fractions, 23 to 36% for IM fractions, 21 to 29% for C fractions, and 9 to 30% for PP fractions. It should be noted that the variability among the PP fractions was negatively affected by the small number of isolated proteins.

### Protein identification and prediction of subcellular localization in isolated fractions.

Our Subcelloc script was used to determine the subcellular localization of the identified proteins. The script used to determine the subcellular localization of C. sakazakii BAA-894 (*in silico*) was also applied to the real sample data for C. sakazakii Cb35.

In three independent subfractionations of C. sakazakii Cb35, a total of 1,801 proteins were identified, of which 31% (558 proteins) were identified by a single peptide (Table 6). A significant number of the single peptide-identified proteins were membrane proteins (30% of OM proteins, 42% of PP proteins, 37% of IM proteins, 36% of MEMBR proteins, and 57% of FLAG proteins). The sequence coverage of these proteins was, on average, less than 5%. This means that even membrane proteins identified by a single peptide are not suitable for inclusion in further analyses. Consequently, only proteins recognized by two or more peptides were included in the total number of identified proteins.

**TABLE 6:** Subcellular localization of all identified proteins of C. sakazakii strain Cb35^*a*^

Protein	Total		EC		FLAG		OM		PP		IM		MEMBR		C		N	
	No.	%	No.	%	No.	%	No.	%	No.	%	No.	%	No.	%	No.	%	No.	%
Total	1,801		14		7		64		45		226		28		1,217		200	
1 peptide	558	31	1	7	4	57	19	30	19	42	84	37	10	36	329	27	92	46
2+ peptides	1,243	69	13	93	3	43	45	70	26	58	142	63	18	64	888	73	108	54

a Total, all identified proteins; EC, extracellular proteins; FLAG, flagellar proteins; OM, outer membrane proteins; PP, periplasmic proteins; IM, inner membrane proteins; MEMBR, membrane proteins; C, cytosolic proteins; N, proteins with unknown localization; No., number of proteins; %, number of proteins in each category expressed as a percentage of the total number of proteins in the category.

**FIG 6:**
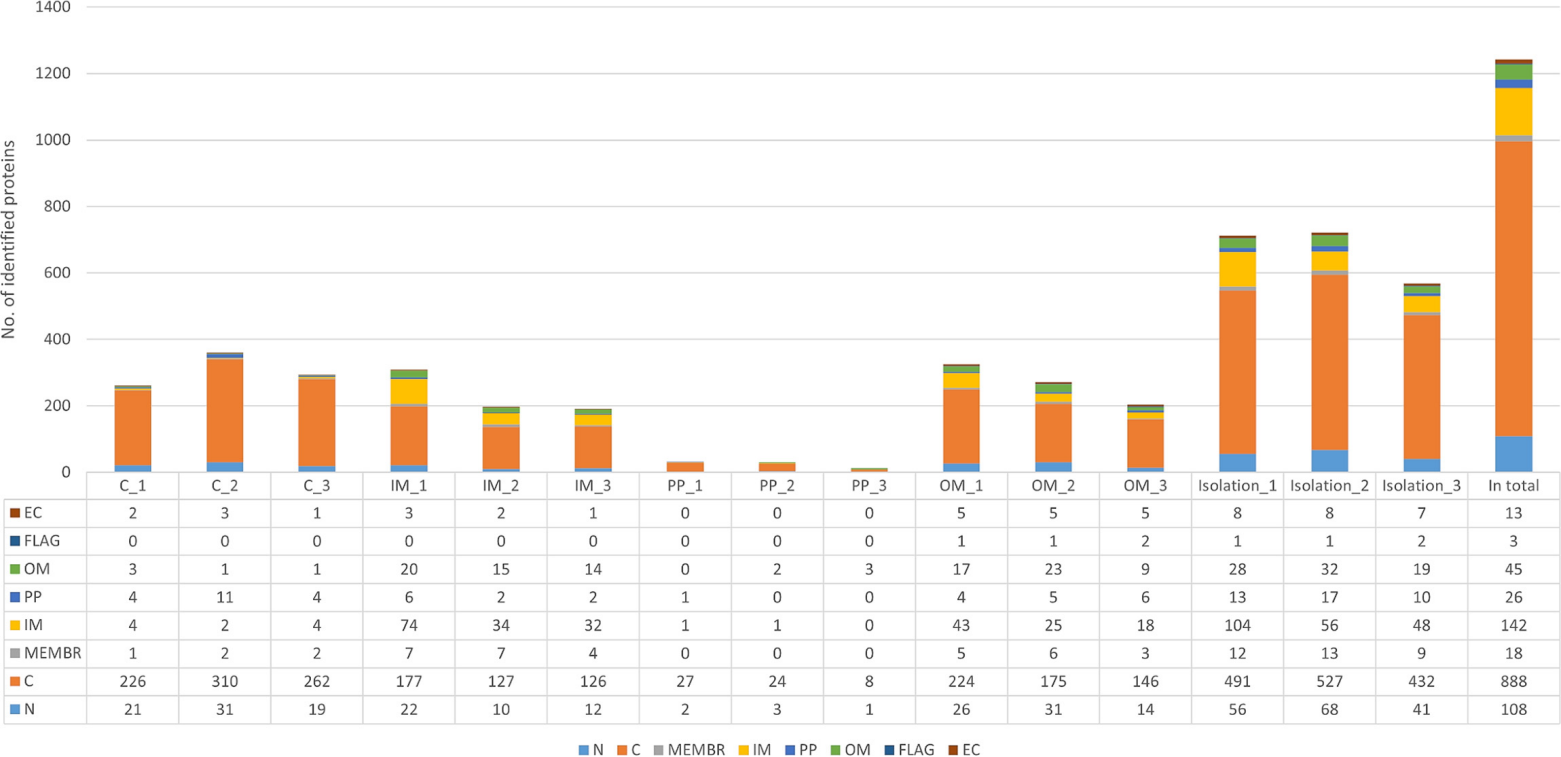
Protein content of isolated fractions in triplicates. EC, extracellular proteins; FLAG, flagellar proteins; OM, outer membrane proteins; PP, periplasmic proteins; IM, inner membrane proteins; MEMBR, membrane proteins; C, cytosolic proteins; N, proteins with unknown localization; C_1/2/3, cytosolic fraction of isolation 1, 2, or 3; IM_1/2/3, inner membrane fraction of isolation 1, 2, or 3; PP_1/2/3, periplasmic fraction of isolation 1, 2, or 3; OM_1/2/3, outer membrane fraction of isolation 1, 2, or 3; Isolation_1/2/3, combined data for all fractions of the corresponding isolation without duplicate proteins; Total, combined data for all isolations without duplicate proteins.

Overall analyses of the proteins identified using two or more peptides showed that C proteins predominated in all subfractions (Fig. 6). Similar amounts of OM proteins were found in both the OM and IM fractions, while the IM proteins dominated in the IM fractions. Flagellar proteins and most EC proteins were found in the OM fractions. The MEMBR proteins, whose localization could not be more specified, were also primarily present in the OM and IM fractions. Very few proteins were identified in the PP fractions, with most of them being cytosolic. Proteins with N localization were evenly distributed across all fractions. Summary data sets were created by merging the proteins identified in all fractions of a particular isolation (1, 2, or 3) and removing duplicate proteins with the same UniParc protein identifiers. Comparable amounts of proteins were identified in the first and second isolations (713 and 722, respectively), while only 568 proteins were identified in the third.

All localization-determined proteins of C. sakazakii Cb35 were compared with the *in silico*-determined ones of C. sakazakii strain BAA-894 (Table 7). According to the UniProt database (2021), the proteome of ATCC BAA-894 consists of 4,421 proteins. A total of 1,243 proteins were identified for Cb35, representing 28% of the predicted proteome. Analysis of the subcellular localization of the predicted proteins revealed that 49% of the predicted C proteins were identified. This high value was because C proteins formed the majority of proteins in all of the isolated subfractions. They consisted of ribosomal proteins and enzymes that process and synthesize substances in the cytosol. The identified IM proteins are typical of the basic proteins involved in cellular metabolism and the cell transport system. However, only 16% of all theoretically expressed IM proteins were identified, possibly because not all proteins are expressed under the growth conditions used. Although the grown cell culture was washed with phosphate-buffered saline (PBS) at the beginning of the protein isolation process (to remove any potentially interfering culture medium residue), 52% of all predicted EC proteins were identified. Most of these proteins are associated with the flagella, which provide cell mobility and the location of which is actually extracellular. Most remarkably, our method could identify 49% of the predicted OM proteins. Many of these proteins are porins (e.g., OmpA, OmpC, OmpF, and OmpX) that serve as channels and may have a role in disease pathogenesis.

**TABLE 7:** Comparison between *in silico*-determined proteins of Cronobacter sakazakii strain BAA-894 and isolated proteins of Cronobacter sakazakii strain Cb35^*a*^

Protein	C. sakazakii strain BAA-894	C. sakazakii strain Cb35	
	No.	No.	%
EC	25	13	52
FLAG	39	3	8
OM	92	45	49
PP	148	26	18
IM	900	142	16
MEMBR	146	18	12
C	1,798	888	49
N	1,273	108	8
Total	4,421	1,243	28

a EC, extracellular proteins; FLAG, flagellar proteins; OM, outer membrane proteins; PP, periplasmic proteins; IM, inner membrane proteins; MEMBR, membrane proteins; C, cytosolic proteins; N, proteins with unknown localization; No., number of proteins; %, number of proteins in each Cb35 category expressed as a percentage of the total number of proteins in the corresponding BAA-894 category.

## DISCUSSION

To identify and characterize proteins from individual cell compartments, it is necessary to have a methodology for determining subcellular localization. However, developing such a methodology is problematic. For all proteomes deposited in the UniProtKB database, localization information is provided under the Proteomes subcategory. For C. sakazakii BAA-894, only a minority (37%) of the proteins included have defined subcellular localization. Although many prediction software tools are freely available, they vary considerably in the effectiveness of their ability to predict subcellular localization. Web-based prediction software designed to determine the protein localization of Gram-negative bacterial species includes NgLOC, MetaLocGramN, CELLO 2.5, and PSORTb 3.0.2 (22–25). Unfortunately, NgLOC (26) and MetaLocGramN (27) were no longer available at the time of our data analysis. CELLO uses the SVM classifier based on multiple feature vectors to determine the localization of all presented proteins (28). The multimodular PSORTb method provides the highest accuracy and, by introducing the category of N localization, does not force prediction when the localization score is below the minimum cutoff (29). In this study, we have introduced the idea of using multiple prediction tools (UniProtKB, CELLO, and PSORTb) to obtain the most accurate information possible about protein localization. For C. sakazakii BAA-894, whose proteome is published in UniProt, these three prediction tools determined the same localization for only 17% (745) of all predicted proteins, which is due to the small number of proteins identified by the UniProtKB tool. When just PSORTb and CELLO are taken into account, the same localization was determined for 60% (2,674) of the proteins (see Fig. S1 in the supplemental material).

Because of the time involved, manually comparing the results from multiple prediction software tools is only possible for a limited number of selected proteins. Thus, we developed a Subcelloc script to process large amounts of data (thousands of proteins) as quickly as possible. Our script automatically compares localization and, importantly, requires at least two software matches to determine the final specific localization. Other proteins for which the prediction tools determine different localizations (not N) are included in the MAN (prediction tools show different results) or MEMBR groups (prediction tools show different results but are associated with membranes).

Proteins vary according to their biological function and localization in the cell. OM and IM proteins primarily consist of β-barrel domain and α-helices, respectively (30). These proteins could be targeted to the membrane by an SP. Therefore, additional web servers (LipoP 1.0 [31], SignalP 5.0 [32], TMHMM 2.0 [33], and BOMP [34]) were tested for the detection of SPs, TMHs, and β-barrel domains. LipoP enables the prediction of SPaseI- and SPaseII-cleaved signal peptides, cytoplasmic proteins, and N-terminal TMH proteins. Although it provides more information in one analysis, LipoP has been surpassed by the recently updated 5.0 version of SignalP, which enables the prediction of not only SPaseI- and II-cleaved signal peptides but also of Tat/SPaseI-cleaved signal peptides. Comparison of the results showed that SignalP could identify more SP-containing proteins than LipoP (data not shown), while TMHMM is suitable for the prediction of TMH motifs, including the number of membrane transitions. To supplement information about unclassified proteins that may be located outside the cytosol, the results from the SignalP and TMHMM web servers were implemented in our script. In addition, BOMP software was tested for the prediction of β-barrel outer membrane proteins. The barrel domain contained a total of 87 proteins, of which the most numerous group was OM proteins (50). This software could provide some extra information, but due to the incompatible way in which it formats results, it was not implemented in our Subcelloc script and not included in further analyses.

For C. sakazakii strain BAA-894, the Subcelloc script (Table 1) could assign 64% (2,833) of all predicted proteins to a specific subcellular category. Of the others, 10% (459) were classified as MAN or MEMBR, with the rest (26%, 1,129) marked as N because the prediction tools did not contain sufficient localization information. Of the 1,129 N proteins, 19 proteins contained a β-barrel domain, 215 proteins contained an SP, and 64 proteins contained at least one TMH. To try to further specify these proteins, the UniProtKB/Swiss-Prot database was used for the BLAST search. However, although it is an expertly curated database with nonredundant protein sequences, this search is a time-consuming nonautomated process that is not guaranteed to determine the localization of all investigated proteins. Thus, only the above-mentioned proteins (757) were used in the BLAST search. Of these, specific subcellular localization was determined for 190 (25%) proteins (Table 2). Only a minority of proteins with a β-barrel domain and an SP were further specified; thus, this group was not included in the BLAST search in our subsequent analyses.

It is generally accepted that OM proteins are coded by about 2 to 3% of the genome of Gram-negative bacteria (35). The Subcelloc script determined 92 of the predicted OM proteins present in the proteome of C. sakazakii BAA-894, which represents 2% of the whole proteome. The fact that this number is on the low side could be the result of several proteins not being correctly assigned as OM due to a lack of information. Approximately 30% of the cell proteome consists of integral membrane proteins (36). The membrane proteins determined by our script (i.e., localization in OM, IM, PP, MEMBR, or FLAG) correspond to 30% of the whole predicted C. sakazakii BAA-894 proteome.

For further LC-MS/MS analysis, it was particularly important to generate a less complex sample by subfractionation. Taking into account the architecture of Gram-negative bacterial cells, these bacteria can be divided into EC, SF, WHC, OM, IM, PP, and C fractions. To separate fractions of the bacterial membrane, various methods have been used, including the use of selective detergents and of ultracentrifugation with detergents or sucrose gradients (15, 18, 37). Thein et al. tested five membrane fractionation methods (numbered 1 to 5) on three strains of Escherichia coli and one strain each of Pseudomonas aeruginosa and Yersinia pseudotuberculosis (37). Three methods enriched only the OM protein fraction while the other two enabled fractionation into OM, IM, PP, and C fractions. Of these, the highest numbers of OM proteins and of non-OM proteins in the OM fraction were observed using method 4. Thus, method 4 was chosen for the subfractionation of C. sakazakii Cb35 cells into C, IM, PP, and OM compartments.

Several studies have used 2DE for the separation of *Cronobacter* membrane proteins, especially OM proteins (11, 16–18). Compared to 1DE, it provides significantly better sensitivity, reproducibility, and individual protein distribution. Despite this, it also has some disadvantages. Only 30 to 50% of all proteins can be visualized, depending on the type of sample. Proteins that are too hydrophobic, of low concentration, or of inappropriate molecular weight are virtually undetectable (38). Typically, membrane proteins are such proteins. Moreover, protein samples containing detergents (e.g., SDS and sarcosyl) cannot be separated by 2D electrophoresis because they are incompatible with isoelectric focusing (39). After protein separation, proteins are usually identified using mass spectrometry. Several approaches have been used for the identification of *Cronobacter* proteins, such as MALDI-TOF MS or LC-ESI-MS/MS (15, 17, 18, 40). However, MALDI is less sensitive and detects significantly smaller amounts of proteins than other sample ionization methods, such as LC-ESI-Q-TOF. Based on these findings, protein separation by 1DE and protein identification by LC-ESI-Q-TOF MS were chosen for this study.

Membrane proteins are soluble in micelle-forming detergent solutions that mimic the natural lipid bilayer of the membrane. These micelles then penetrate the membrane, eventually disrupting it and enabling protein dissolution (41). For all detergents selected in this work (anionic, zwitterionic, and nonionic), the CMC was achieved at a concentration of 0.5% (Table S1), but the anionic detergents, sarcosyl and SDS, were most efficient in protein dissolution (Fig. 2). This was also confirmed by using the BCA method to determine concentration (Table 4). In some studies, sarcosyl has been used as a detergent to isolate the sarcosyl-insoluble fraction (40, 42–44), but before we used it here, we first needed to make sure that our method does not lead to the loss of sarcosyl-insoluble proteins. Therefore, various sample preparation methods were tested involving centrifuged samples (supernatant [S] and pellet [P]) and a noncentrifuged sample (whole [W]). Sample preparation without centrifugation identified the fewest proteins, making W unsuitable for the subsequent analyses. After centrifugation, unique proteins were identified in both S and P samples. More unique OM proteins were identified in P (10 compared to 6 in S), but analysis of the supernatant identified the largest number of proteins (506) and membrane proteins (100), so S was chosen as the sample preparation method for the following analyses.

In mass spectrometry, the repeatability of analyses of highly complex samples presents significant challenges. Integral membrane proteins are amphipathic and contain more hydrophobic regions. These regions have fewer arginines and lysines (or they are hidden in the membrane) and form longer fragments after trypsin digestion (7, 45). In addition, some membrane proteins are known to contain intramolecular amide bonds, making them difficult to detect by mass spectrometry (46). Furthermore, strongly hydrophobic peptides are difficult to extract from the gel and can also remain bound to the reverse phase of the microcolumns used for the desalination and concentration of the samples prior to MS/MS (14). Thus, it was desirable to determine the repeatability of sample preparation, in-gel digestion, and LC separation with random peptide fragmentation on MS. LC-MS/MS had the most impact on sample repeatability. In tandem mass spectrometry, this phenomenon mainly occurs in complex samples, where a different set of peptides can be selected for fragmentation when the analysis is repeated and, thus, a different protein identified (6, 39, 47, 48). Despite our use of sample subfractionation to reduce complexity, each sample still contained a large number of peptides with similar retention times. Consequently, signal overlap and peptide suppression can occur due to the small number of peptides from less abundant membrane proteins (39, 47). Although these technical challenges might be overcome by using MS instrumentation with higher sensitivity and resolution, or by adding another chromatographic dimension (e.g., a strong cation exchange column) (38, 49), such approaches would be even more costly and time-consuming. Another issue is that measurement may be affected by the time lag between injections because measuring samples in random order can lead to the minor evaporation and probable adsorption of several peptides on the tube walls (50). This is why we repeated the subfractionation three times independently. Furthermore, due to this low repeatability and our aim to describe the whole proteome, all of the data obtained were combined into one data set.

In this work, a total of 1,801 proteins were identified across all isolated subfractions of C. sakazakii Cb35. Because the identification of proteins by a single peptide is unreliable, each protein was required to contribute two different peptide sequences in a single LC-MS/MS analysis to be counted as detected (47). The proteins identified by just a single peptide corresponded to 31% of all identified proteins; thus, 1,243 proteins were used for subsequent analyses. There was significant cytosolic protein contamination in all analyzed fractions, which was expected as this is known to occur during cell lysis and is practically impossible to avoid (37). The low abundance of proteins in the PP fraction may have been due to increased protein transfer to other fractions, with the periplasmic proteins integrally or peripherally bound to the outer side of the spheroplast IM or to the inner side of the released OM. The larger amount of OM proteins in the IM fractions can be explained by the addition of Triton X-100 detergent during the subfractionation process, which was sufficient to dissolve these proteins and transfer them to the supernatant.

Overall analyses of the fractionated C. sakazakii Cb35 cells led to the identification of 1,243 proteins, which corresponds to a notable 28% of all predicted proteins of C. sakazakii BAA-894. Of this total, the subcellular localization of 234 proteins was determined to be membrane associated. Remarkably, 45 OM proteins, representing 49% of all predicted OM proteins, were also identified. Thein et al. used method 4 for E. coli subfractionation, analyzing only the isolated OM fraction and identifying a total of 31 OM proteins (37). In our study, a significant number of OM proteins were identified in the IM fraction, indicating the need to analyze both the OM and IM fractions. Carranza et al., using another type of subfractionation method (EC, SF, and WHC fractions) with 1DE-LC-ESI-MS/MS and 2D-LC-MALDI-TOF/TOF, identified 19% of all predicted C. sakazakii BAA-894 proteins (22 OM, 156 membrane associated) (15). They also counted proteins identified by just one peptide in at least two isolations. Thus, 28% of all predicted proteins represents a significant improvement and shows that our combined subfractionation technique and sample processing methodology provide better total proteome mapping.

In conclusion, to our best knowledge, we have provided the first study of the subfractionation of C. sakazakii cells, indeed of the cells of any *Cronobacter* strain, into four compartments. Having determined the best detergent and sample processing procedure, most attention was focused on the protein content of the OM fraction. Our Subcelloc script for analyzing the subcellular localization of large data sets determined 1,243 proteins, of which 45 were OM proteins. This is significant because OM proteins are an important component of the cell wall and are directly involved in host-pathogen interactions. Thus, our approach provides greater insight into the expressed OM proteins, which can help to clarify their involvement in virulence mechanisms. Indeed, to identify the biomarkers that enable us to differentiate between the virulence of the various species in this genus, we have already begun to apply our method and script to the analyses of all other *Cronobacter* species.

## MATERIALS AND METHODS

### Bacterial strain and growth conditions.

Cronobacter sakazakii strain Cb35, clinically isolated from spinal fluid, was used. This strain has been characterized in previous publications: its lipopolysaccharide composition is type S O:2 (51); its restriction fragment length polymorphism profiles (*rpoB*-PCR-RFLP) are C1, H1, and M5 (52); and its MLST sequence type is 4 (data not published). This sequence type belongs to the strains of clonal complex 4, which are associated with neonatal infections (53). To isolate the proteins, the bacteria were grown for 12 h at 37°C in tryptic soy broth medium under constant stirring (100 rpm).

### Subfractionation.

The subfractionation methodology was based on the literature (37) but adapted to our laboratory conditions. In general, the grown cells were collected at 6,000 × *g* for 30 min at 4°C. The formed pellet was washed with 0.01 mol liter^−1^ PBS at pH 7.4 and resuspended in buffer 1 (pH 8; 0.2 mol liter^−1^ Tris-HCl, 1 mol liter^−1^ sucrose, 1 mmol liter^−1^ EDTA, 1.5 mg mL^−1^ lysozyme), and the suspension was incubated for 5 min at room temperature (RT). Sterile water was added and the mixture incubated for 20 min on ice, leading to the formation of spheroplasts. The suspension was ultracentrifuged at 200,000 × *g* for 1 h at 4°C, the supernatant containing the PP fraction was collected, and the pellet with spheroplasts was resuspended in buffer 2 (pH 7.5; 10 mmol liter^−1^ Tris-HCl, 5 mmol liter^−1^ EDTA, 0.2 mmol liter^−1^ dithiothreitol [DTT]). The spheroplasts were lysed by sonication (15 W for 10 min on ice). The unlysed cells were spun at 5,500 × *g* for 10 min at 4°C and discarded. The supernatant then was ultracentrifuged at 300,000 × *g* for 4 h at 4°C. The supernatant containing the C fraction was collected, and the remaining pellet with membranes was resuspended in buffer 3 (pH 8; 50 mmol liter^−1^ Tris-HCl, 2% [vol/vol] Triton X-100, 10 mmol liter^−1^ MgCl_2_). The membrane suspension was centrifuged at 85,000 × *g* for 30 min at 4°C, the supernatant containing the IM fraction was collected, and the pellet with the OM fraction was washed 1× with buffer 3 followed by 3× with sterile water. All isolated fractions (PP, C, IM, and OM) were lyophilized and stored at −80°C. This subfractionation method was repeated three times independently.

### Preparation of isolated fractions for further analysis.

The lyophilized PP, C, and IM fractions were dissolved in sterile water at a final concentration of 1 mg mL^−1^. These fractions were vortexed for 30 min at RT before being centrifuged at 85,000 × *g* for 30 min at 4°C. The supernatant was either used immediately or stored at −80°C.

To dissolve the lyophilized OM fraction, six detergents (SDS, sarcosyl, DOC, CHAPS, Triton X-100, and Tween 20) were tested at concentrations of 0.5% and 1%. The OM fraction was resuspended in PBS with a specific detergent at a concentration of 1 mg mL^−1^. The suspension was homogenized in a Potter-Elvehjem homogenizer and vortexed for 30 min at RT.

Three techniques of OM sample preparation were tested. The OM fraction was resuspended in 1 mL of PBS with 0.5% sarcosyl at a concentration of 1 mg mL^−1^, homogenized, and vortexed for 30 min. This sample then was divided; half was used as sample W, and the other half was centrifuged at 85,000 × *g* for 30 min at 4°C. The supernatant (S) was collected and stored; the pellet (P) was resuspended in 50 μL of reducing Laemmli buffer and incubated for 30 min at RT with occasional stirring. All three samples (W, S, and P) were prepared in independent duplicates, and each sample was injected twice into an LC column and analyzed by LC-MS/MS.

### Determination of protein concentration.

The protein concentration was determined using the Pierce BCA protein assay kit. Due to the high sucrose content in buffer 1, the PP fraction had to be dialyzed against PBS with an Amicon Ultra-0.5 centrifugal filter prior to the determination.

### SDS-PAGE.

The protein composition of the dissolved samples was analyzed by Laemmli–SDS-PAGE. Briefly, the samples were mixed with Laemmli buffer at a ratio of 5:1 under denaturing conditions and incubated for 10 min at RT. The samples were loaded into a stacking gel (4%) and fully separated in a separating gel (12%). The gels were Coomassie stained.

### In-gel digestion and peptide extraction.

The procedure used for protein digestion was adapted from the literature (54). The samples were mixed with Laemmli buffer at a ratio of 5:1 under denaturing conditions and incubated for 10 min at RT. Subsequently, the samples were separated by SDS-PAGE to a depth of only 1 cm of the separating gel, stained with Coomassie for 30 min, and then washed in water three times for 10 min. The protein zone was excised from the gel, cut into cubes (1 mm by 1 mm), washed with water, and dried with acetonitrile (ACN) solution. The proteins were then reduced with DTT solution (10 mmol liter^−1^) for 45 min at 56°C and alkylated with iodoacetamide solution (55 mmol liter^−1^) for 30 min at RT in the dark. The cubes were subsequently washed and dried with ACN solution, and the contained proteins were digested by trypsin (12.5 ng μL^−1^) at 37°C overnight. After incubation, the peptides were extracted from the gel with ACN solutions (35%, 70%). The solvent was removed by vacuum evaporation, and the peptides were dissolved in 0.1% trifluoroacetic acid. The samples containing the isolated peptides were desalted on a C_18_ ZipTip microcolumn according to the manufacturer's protocol. The desalted samples were stored at −20°C prior to mass spectrometric analysis.

### Mass spectrometric analysis.

Mass spectra were obtained using a Dionex Ultimate 3000 RSLC nano-ultrahigh-performance LC system coupled with an ESI-Q-TOF Maxis Impact mass spectrometer. The desalted samples were dissolved in water-ACN-formic acid solution (96.9:3:0.1) and subsequently applied to an Acclaim PepMap 100 C_18_ column (100 μm by 2 cm; particle size, 5 μm) with a mobile phase (0.1% formic acid in 3% ACN) at a flow rate of 5 μL min^−1^ for 5 min. The peptides were eluted from the capture column on an Acclaim PepMap RSLC C_18_ analytical column (75 μm by 150 mm; particle size, 2 μm) with a mobile phase (0.1% formic acid in ACN) using a linear gradient of 3 to 35% of ACN in 30 min; they were directly eluted into the ion source (captive spray). Measurements were performed in positive ion mode with a selection of precursor ions ranging from 400 to 1,400 *m/z*; up to 10 precursor ions were selected for fragmentation from each mass spectrum. Peak lists were obtained from the raw data using Data Analysis version 4.1 and uploaded to the Proteinscape bioinformatics platform for managing proteomics data. Mascot server version 2.4.1 with a search in the *Cronobacter* database (see “Bioinformatics,” below) was used to identify the proteins. The search parameters were the following: enzyme used, trypsin; one missing cleavage allowed; tolerance, 10 ppm in MS mode and 0.05 Da in MS-MS mode; fixed carbamidomethylation of cysteine; variable oxidation of methionine. The results were filtered so that the false discovery rate was 1%. All samples related to sample preparation testing and variability determination were analyzed in duplicate unless otherwise stated.

### Technical repeatability.

To evaluate the repeatability of our protein identification method, the influences of sample preparation, in-gel digestion, and LC-MS/MS were tested. Briefly, the same amount (1 mg) of the OM fraction was dissolved three times (samples 1, 2, and 3) under the same conditions. Sample 1 was applied to the polyacrylamide gel three times (samples A, B, and C) and to samples 2 and 3 only once. The protein zones were excised from the gel and digested with trypsin. Samples A, B, and C were each analyzed by LC-MS/MS twice (samples I and II).

### Bioinformatics. (i) Database.

Mass spectrometry identification of the isolated proteins was performed using a protein database created from proteomes available in the UniProtKB database as of June 2021. To cover a sufficiently wide range of proteins, the database included the following 18 *Cronobacter* proteomes (a total of 77,383 protein sequences): C. sakazakii BAA-894, C. sakazakii 701, C. sakazakii 696, C. sakazakii MOD1_2011-18-05-03, C. sakazakii MOD1_CQ32, C. sakazakii MOD1_GK964, C. sakazakii GZcsf-1, C. sakazakii 5563_17, *C. dublinensis* LMG 23823, *C. dublinensis* 1210, *C. dublinensis* 582, *C. condimenti* 1330, *C. malonaticus* S1, *C. malonaticus* 45402, *C. universalis* NCTC 9529, *C. turicensis* DSM 18703, *C. turicensis* MOD1-Sh41s, and *C. muytjensii* MOD1-Md1s.

### (ii) Data sets.

The proteins were compared using UniParc protein identifiers. These identifiers were used rather than UniProtKB accession numbers because accession numbers can be deleted from UniProtKB during database cleanup. Moreover, a UniParc identifier refers to the unique sequence of a protein in the UniProtKB database, while an accession number refers only to a unique protein within the bacterial strain. Proteins with the same UniParc protein identifiers but with a lower identification score, or identified by either a single peptide or fewer peptides than their counterparts, were removed from the data set. This means that only proteins identified by at least two unique peptides were considered in the analyses.

### (iii) Subcellular localizations.

The subcellular localization of the proteins was determined by a Subcelloc script developed for this study. Our script uses localization information from the free web tools UniProtKB, PSORTb 3.0.2, and CELLO 2.5 to evaluate the most probable subcellular localization as a combination of at least two identical results from these tools. The protein groups MAN and MEMBR were implemented in the script for proteins that were hard to categorize. Further information is input to the script by SignalP 5.0 and TMHMM 2.0, which, for proteins with N localization, determine the presence of SP or TMH, respectively. The sequences of proteins from the MAN, MEMBR, and N with TMH groups were subsequently compared to determine the final localization. These proteins then were compared with similar proteins from closely related genera using BLAST search on the UniProt website using the UniProtKB/Swiss-Prot database. More information about and exact instructions for our Subcelloc script and its use in the data evaluation can be found in a user guide on the website https://github.com/Novotnye/The_Subcelloc_script.

### (iv) Statistics.

The similarity between the isolated fractions was analyzed with BioNumerics 7.5 software. The dendrograms were calculated using the Jaccard correlation with a maximum limit value. The clustering method used was unweighted pair-group arithmetic mean (UPGMA) with Euclidean distance.

To compare various data sets, Venn diagrams were created using InteractiVenn software. UniParc protein identifiers were loaded into the sets.
