# Supplementary material for: Membrane Proteins and Proteomics of Cronobacter sakazakii Cells: Reliable Method for Identification and Subcellular Localization
Source: Appl Environ Microbiol. 2022 Apr 18;88(9):e02508-21. doi: 10.1128/aem.02508-21 (PMC9088360; doi:10.1128/aem.02508-21)
Supplement: Supplemental file 1 — Tables S1 and S2 and Fig. S1. Download aem.02508-21-s0001.pdf, PDF file, 0.4 MB [file aem.02508-21-s0001.pdf]

Table S1 Tested detergents and their critical micelle concentration

**Detergent**	**Type**	**CMC [mmol l^{-1}]**	**CMC of 0.5% solution [mmol l^{-1}]**	**CMC of 1% solution [mmol l^{-1}]**
Lauroylsarcosine	anionic	14.6	17	34
SDS	anionic	7-10	17.3	35
DOC	anionic	2-6	12.1	24
CHAPS	zwitterionic	6-10	8.1	16
Triton X-100	nonionic	0.2-0.9	8.0	16
Tween 20	nonionic	0.06	4.1	8

Lauroylsarcosine - N-Lauroylsarcosine sodium salt; SDS - sodium dodecyl sulfate; DOC - sodium deoxycholate; CHAPS - (3-((3-cholamidopropyl) dimethylammonio)-1-propanesulfonate; CMC - critical micelle concentration

Table S2 Protein concentration determined using the Pierce-BCA Protein Assay Kit for samples of isolated fractions

Fractions	Protein concentration [μg ml^{-1}]
OM_1	563±8
OM_2	535±1
OM_3	489±9
IM_1	103±5
IM_2	124±3
IM_3	112±6
PP_1	17±1
PP_2	17±1
PP_3	14±1
C_1	209±6
C_2	232±4
C_3	134±1

OM_1/2/3 - outer membrane fraction in the corresponding isolation; IM_1/2/3 – inner membrane fraction in the corresponding isolation; PP_1/2/3 – periplasmic fraction in the corresponding isolation; C_1/2/3 – cytosolic fraction in the corresponding isolation

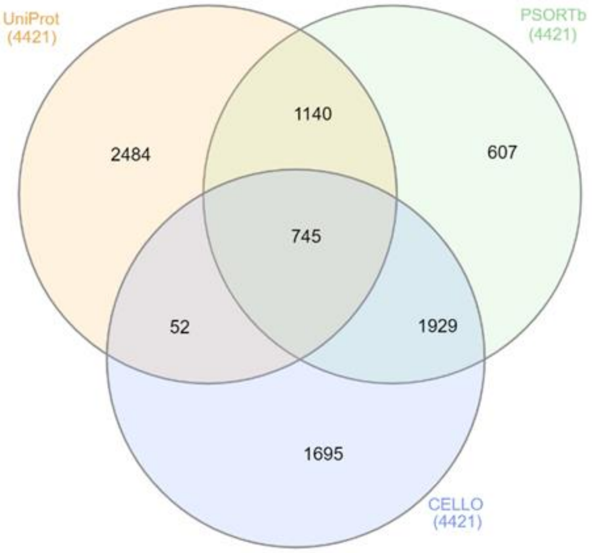

Figure S1 Venn diagram representing the predicted subcellular localization using PSORTb, CELLO, and UniProtKB
